# SiSTL2 Is Required for Cell Cycle, Leaf Organ Development, Chloroplast Biogenesis, and Has Effects on C_4_ Photosynthesis in *Setaria italica* (L.) P. Beauv.

**DOI:** 10.3389/fpls.2018.01103

**Published:** 2018-07-30

**Authors:** Shuo Zhang, Sha Tang, Chanjuan Tang, Mingzhao Luo, Guanqing Jia, Hui Zhi, Xianmin Diao

**Affiliations:** Institute of Crop Sciences, Chinese Academy of Agricultural Sciences, Beijing, China

**Keywords:** dCMP deaminase, chloroplast biogenesis, cell cycle, cell expansion, C_4_ photosynthesis, *Setaria italica*

## Abstract

Deoxycytidine monophosphate deaminase (DCD) is a key enzyme in the *de novo* dTTP biosynthesis pathway. Previous studies have indicated that DCD plays key roles in the maintenance of the balance of dNTP pools, cell cycle progression, and plant development. However, few studies have elucidated the functions of the DCD gene in Panicoideae plants. *Setaria* has been proposed as an ideal model of Panicoideae grasses, especially for C_4_ photosynthesis research. Here, a *Setaria italica* stripe leaf mutant (*sistl2*) was isolated from EMS-induced lines of “Yugu1,” the wild-type parent. The *sistl2* mutant exhibited semi-dwarf, striped leaves, abnormal chloroplast ultrastructure, and delayed cell cycle progression compared with Yugu1. High-throughput sequencing and map-based cloning identified the causal gene *SiSTL2*, which encodes a DCD protein. The occurrence of a single-base G to A substitution in the fifth intron introduced alternative splicing, which led to the early termination of translation. Further physiological and transcriptomic investigation indicated that *SiSTL2* plays an essential role in the regulation of chloroplast biogenesis, cell cycle, and DNA replication, which suggested that the gene has conserved functions in both foxtail millet and rice. Remarkably, in contrast to DCD mutants in C_3_ rice, *sistl2* showed a significant reduction in leaf cell size and affected C_4_ photosynthetic capacity in foxtail millet. qPCR showed that *SiSTL2* had a similar expression pattern to typical C_4_ genes in response to a low CO_2_ environment. Moreover, the loss of function of *SiSTL2* resulted in a reduction of leaf ^13^C content and the enrichment of DEGs in photosynthetic carbon fixation. Our research provides in-depth knowledge of the role of DCD in the C_4_ photosynthesis model *S. italica* and proposed new directions for further study of the function of DCD.

## Introduction

Deoxycytidine monophosphate deaminase (DCD) is a key enzyme in the *de novo* deoxythymidine triphosphate (dTTP) synthesis pathway. DCD catalyzes the deamination of deoxycytidylate (dCMP) to produce deoxyuridine monophosphate (dUMP), the latter of which is the substrate that ultimately forms dTTP (Ellims et al., 1981). As described in previous studies, DCD contains two conserved motifs, HXE and PCXXC, which reportedly function as a zinc-binding motif (Weiner et al., 1993). The DCD monomer is characterized to have between four and five α-helixes and six β-sheets, which are arranged in an α-β-α sandwich (Scortecci et al., 2017). Six DCD monomers combine to form a biologically active homohexamer in bacteria and animals (Hou et al., 2008). As an allosteric enzyme, DCD contains an allosteric site. dTTP and deoxycytidine triphosphate (dCTP) are the inhibitor and activator of DCD, respectively, which competitively bind to the allosteric site to control protein stability and further regulate the activity of DCD (Hou et al., 2008; Marx and Alian, 2015). This allosteric regulation depends on the participation of divalent metal ions, such as Ca^2+^ and Mg^2+^ (Scortecci et al., 2017). The pools of dTTP and deoxyribonucleoside triphosphates (dNTPs) are basic elements essential for DNA synthesis, replication, recombination, and repair (Kumar et al., 2011), which further guarantee the normal procession of these biological pathways. Two enzymes, the abovementioned DCD and ribonucleoside reductase (RNR), are mostly responsible for the maintenance of sufficient dTTP (Ke et al., 2005). RNR, which catalyzes the reduction reaction to produce deoxy-ribonucleotide diphosphate (dNDPs), is responsible for the *de novo* biosynthesis of four dNTPs to ensure the dNTP pools balance (Buckland et al., 2014). In addition to the *de novo* pathway, dTTP can be supplemented by the thymidine kinase (TK) salvage pathway in a majority of higher eukaryotes. TK, as the key enzyme in the salvage pathway, can catalyze the phosphorylation of deoxidized thymidine to form deoxythymidine monophosphate, the substrate to produce dTTP (Leija et al., 2016). When either of the *de novo* or salvage pathway is defective, the other can compensate for the loss of pyrimidine synthesis function (Leija et al., 2016). Thus, these pathways are mutually interrelated to maintain sufficient dTTP and the balance of the dNTP pools. In addition, the biosynthesis of dTTP is a highly coordinated process.

Deoxycytidine monophosphate deaminase defects always lead to the imbalance of the dNTP pools and further cause the abnormal synthesis of DNA. In the process of DNA synthesis, the accuracy of DNA polymerase depends on the supply of dNTPs (Buckland et al., 2014). When the correct dNTP inserts into a nucleotide chain, it competes with three other kinds of dNTPs. In addition, the fidelity of DNA replication also depends on the GC% in the dNTP pools. Thus, unbalanced dNTP pools increase the probability of the insertion of the wrong base (Gu and Li, 1994; Gawel et al., 2014). In addition, unbalanced dNTP pools can increase the frequencies of insertion/deletion mutations (Kumar et al., 2011). It has been reported that in fission yeast, the deletion of *DCD1* leads to drastic changes in the accumulations of dCTP and dTTP. The mutant strain becomes sensitive to DNA damage inducers, has a decreased capacity for DNA replication and repair, even appears the collapse of replication folk (Sanchez et al., 2012). Previous studies also reported that in budding yeast, *DCD* or *RNR* mutation could increase the mutation rates, slow down the DNA replication, and impact the genome integrity and stability (Kohalmi et al., 1991; Sanchez et al., 2012). In rice, the mutation of *OsDCD* results in DNA damage and leaf cell apoptosis (Niu et al., 2017).

In contrast, DCD decrease sometimes impaired cell cycle progression. The S-phase is the period in which the majority of DNA replication is established. Thus, the adequacy of dNTPs is a precondition of entrance into S-phase (McIntosh et al., 1986). Insufficient dNTPs or the imbalance of dNTP pools can activate the S-phase checkpoint, delay the progress of the cell cycle, decrease the cell proliferation rate, and even lead to cell apoptosis (Kumar et al., 2010). Previous studies have established that DCD or RNR deficiency in yeast resulted in cell cycle arrest in the S-phase (Elledge and Davis, 1990; Sanchez et al., 2012). The deficits of DCD in rice also cause cell cycle progression defects (Niu et al., 2017). DCD and other dNTPs biosynthetic genes are coordinated with cell cycle progression. Some cell cycle associated enzymes participate in the regulation of nucleotide metabolizing genes (Ke et al., 2005). In addition, the activities of most dTTP synthetic enzymes exhibit periodic fluctuations during the cell cycle (McIntosh et al., 1986). TK and thymidylate kinase (TMPK) are the targets of the cell cycle proteasome APC/C and can be degraded by APC/C during the G1-phase to maintain the balance of the dNTP pools in mammals (Ke et al., 2005). E2Fs, as important cell cycle associated transcription factors (TFs), can induce specific *RNR1a* expression in the G1/S-phase (Lincker et al., 2004). However, *DCD1* is reported to have a periodic expression level during the cell cycle in HeLa cells, but is transcribed at a constant level in *Saccharomyces cerevisiae* (McIntosh et al., 1986).

In higher plants, mutations of dNTPs synthesis genes always result in abnormal leaf development. For example, the phenotype of the *Arabidopsis thaliana RNR1* mutant has crinkled leaves with white pits. The mutant has fewer, larger, and dysplastic chloroplasts (Garton et al., 2007). Similarly, mutations in large and small submits of RNR in rice lead to dwarf plants, white stripe leaves, low chlorophyll contents, and undeveloped chloroplasts (Yoo et al., 2009; Qin et al., 2017). There are few reports on DCD gene functions in higher plants. A previous study showed that rice *OsDCD* mutant *st2* has white stripe leaves and reduced plant height. The chloroplast development and chlorophyll accumulation of *st2* are defected (Xu et al., 2014). In a recent study, another rice *DCD* mutant, *alr*, showed similar, but more severe phenotypes compared with *st2*, which had a small grain size and necrotic spots on the leaves. In addition, the cells of the *alr* mutant were increased in size, but decreased in number (Niu et al., 2017). Previous studies have proposed several explanations for these phenotypes in the *OsDCD* mutant. First, the unbalanced dNTP pools impaired the plastid genome replication and delayed chloroplast development. Second, the cell cycle progression and cell division were influenced, which resulted in small organs and dwarf plants (Niu et al., 2017).

Here, we isolated an *Setaria italica* mutant *sistl2* from EMS-induced lines of the Yugu1 cultivar. The mutant gene *SiSTL2* encodes a DCD protein. *sistl2* has similar phenotypes to those of *alr* and *st2* in rice. More interestingly, we found that *sistl2* was different to the rice *alr* mutant, including significantly reduced leaf cell size, normal second and third leaves, and uninfluenced grain size. It is worth noting that the C_4_ photosynthetic character of *sistl2* was affected, which has not been previously reported. In our study, we analyzed the mutant phenotypes, the expression of the causal genes, and expounded the function of *SiSTL2* in *S. italica*. Our research provides further insights into the role of DCD in the C_4_ model *S. italica* and proposed new perspectives for further study of DCD functions.

## Materials and Methods

### Plant Materials and Growth Condition

The *sistl2* mutant was obtained from the EMS-induced *S. italica* cultivar Yugu1. The identified mutant plants were backcrossed with Yugu1 three times before the characterization of the morphological and physical traits. To measure the agronomic traits and to determine the chlorophyll content and photosynthesis rate of Yugu1 and *sistl2*, the plants were grown in the experimental fields of the Institute of Crop Sciences, Chinese Academy of Agricultural Sciences, in Beijing (116.6° E, 40.1° N), China.

### Chlorophyll Content, Photosynthetic Rate, and Chlorophyll Fluorescence

*Setaria italica* leaves harvested from the heading stage were cut into pieces and soaked in 95% alcohol for approximately 3 days until the leaf pieces were discolored completely. The supernatant was then collected and the absorbance values at 665 and 649 nm were measured by using a UV-1800 ultraviolet/visible light spectrophotometer. Chlorophyll *a* (Chl *a*) and chlorophyll *b* (Chl *b*) levels were then calculated by using the equation reported by Lichtenthaler (1987). The photosynthetic parameters were measured on fine mornings by using the Li-6400 portable photosynthesis system (LI-COR, Lincoln, NE, United States) to analyze mature leaves from five individual heading stage plants (6400-02B LED light source, ParIn = 1000 μmol m^-2^ s^-1^). The ΔΦPS II [(Fm′–Fs)/Fm′] was calculated from the equation mentioned by Hu et al. (2014).

### Phenotypic Analysis of Leaf Venation Patterns

The leaves gained from the heading stage were observed and photographed by using an Anyty^TM^ 3R MSV500 portable digital microscope (3R Eddytek, Beijing, China). For the I_2_–KI dye, the leaves were cut into pieces of 5 mm × 5 mm and fixed in FAA (3.7% formaldehyde, 50% ethanol, and 5.0% glacial acetic acid) overnight at 4°C, followed by a graded ethanol series and xylene series, and finally dyed in I_2_–KI for 12 h. The samples were then observed and photographed by using a light microscope. For the cell size measurement, 20 medium-sized cells from three horizons were analyzed by using Image-Pro plus 6.0 (Media Cybernetics, Silver Spring, Georgia Avenue, United States).

### Leaf Microstructure Observations

For the leaf cross section, the leaves of the seedling stage were cut into pieces of 2 mm × 1 mm, fixed, and treated as described above. After the treatment, the samples were embedded in resin. Microtome sections were stained with toluidine blue and photographed by using a light microscope.

For transmission electron microscopy (TEM) analysis, the leaf materials were obtained from seedlings of *Yugu1* and *sistl2* and cut into pieces of 2 mm × 1 mm. The materials were fixed overnight in 0.1 M phosphate buffer with 2.5% glutaraldehyde. Then the samples were washed three times with 0.2 M phosphate buffer and post-fixed in 1% osmium tetroxide for 1 h. After staining with uranyl acetate, the samples were further dehydrated in a gradient ethanol series and finally embedded into resin. Ultrathin sections were made and examined by using a JEM 1230 TEM.

### Map-Based Cloning and High-Throughput Sequencing

For MutMap+ analysis (Fekih et al., 2013), the leaf samples were harvested from recessive individuals and dominant individuals, respectively. Then, two DNA pools contained the same amounts of DNA samples from 27 recessive/dominant individuals for whole genome resequencing were built. The resequencing was performed by using the Illumina HiSeq 2500 platform with a 150 bp paired-end strategy. The raw sequencing data was deposited at EMBL-EBI in the European Nucleotide Archive database under the accession number ERP106777. Data from the two sample pools were aligned to each other and analyzed in accordance with methods described in our previous study (Xiang et al., 2017).

To exclude irrelevant SNPs from the results of MutMap+ analysis, an F_2_ population from the hybridization between *sistl2* and the SSR41 cultivar was used for mapping of the SiSTL2 locus. Three SSR markers based on earlier studies (Jia et al., 2009; Zhang et al., 2014) were adopted and an In-Del marker Ins9-1 was designed for gene cross positioning (**Supplementary Table S1**). To verify the change in transcript splicing, coding sequences of *Seita.9G511200* in WT and *sistl2* were amplified by using PCR primers (5′-ATGGCCTCGACGAGGGA-3′ and 5′-AAGGGCAAGGGAGTGAT-3′) and transformed into vectors by using the pEASY-Blunt cloning Kit (TRAN, Beijing, China). Four clones of each transformed cases were picked for sequencing by using the M13+/- universal primer.

### Complementation Assay

The PCR reaction for the amplification of the promoter (primer with underlined in-fusion adaptor: 5′-GGCCAGTGCCAAGCTTCGTCGTTCCCTCCAAGTT-3′ and 5′-TCGAGGCCATGGATCCCGCGCTCGGGCGGTGGGGA-3′) and genome sequence (primer with in-fusion adaptor underlined: 5′-CCCGAGCGCGGGATCCATGGCCTCGACGAGGGA-3′ and 5′-GATCGGGGAAATTCGAGCTCAGAGGATCAAGCCATAGGACAC-3′) of *SiSTL2* were performed and transformed into the pTCK303 vector by using the In-Fusion HD Cloning Kit (Cat no. 072012, Clontech, United States) in two separate operations. The rice *Ubiquitin* gene was used as a reference for the RT-qPCR of transgenic lines in accordance with a previous report (Wang Y. et al., 2017).

### Bioinformatics Analysis

The sequence, structure, and function annotation of the candidate genes were obtained from the *S. italica* V2.2 database of *Phytozome*^1^. The functional domains and motifs were predicted from the amino acid sequence by Conversed Domain search in *NCBI*^2^. The 3D structure analysis was produced by the *PDB* database^3^. Transcription factor binding sites were predicted by PlantRegMap^4^.

### Enzymatic Assays

For enzymatic assays, Wild-type *SiSTL2* cDNA and mutant *SiSTL2* cDNA (sequence before the mutant site) was subcloned into pCold vector and transformed into *E. coli* Transetta (DE3) for prokaryotic expression (sequences of primers with underlined in-fusion adaptor: for WT, 5′-TACCCTCGAGGGATCCATGGCCTCGACGAGGGA-3′ and 5′-GCTTGAATTCGGATCCTGGCTCCTGAAACTTGATCG-3′; for mutant, 5′-TACCCTCGAGGGATCCATGGCCTCGACGAGGGA-3′ and 5′-GCTTGAATTCGGATCCTAGGATTATTCCCTCTTGGCT-3′). Bacterial cultures (2 ml) were grown overnight at 37°C in LB medium, then transformed into 200 ml LB, and cultivate until OD_600_ = 0.6. Then the cells were induced using 0.3 mM isopropyl β-Dthiogalactopyranoside (IPTG) for 10 h at 15°C. Next, the cells were harvested by centrifugation and resuspended in 1 × PBS buffer, containing 1 mM PMSF and 100 unit/ml DNase. After disruption by sonication, the cell debris was removed by centrifugation at 16,100 *g* for 10 min. The soluble lysis fraction was purified by histidine specific Ni-NTA agarose resin and the target protein was eluted in elution buffer. Finally, the wild-type and mutant fusion protein with histidine tag were detected by SDS-PAGE, and used in enzyme assays.

Enzyme assays were conducted to measure the specific activity at 30°C in the buffer described previously with 100 μM dCTP and 0.2 mM MgCl_2_ (Hou et al., 2008; Niu et al., 2017) by detecting the decrease in absorption at 290 nm using a UV-1800 ultraviolet/visible light spectrophotometer. The dCMP concentration varied from 0 to 3 mM with a grade of 0.5 mM. The enzyme concentration was fixed at 0.5 μg/ml. All measurements were performed three times, and average values and standard deviation were calculated.

### qRT-PCR Analysis

The total RNA was gained from fresh plant tissues by using a Pure Link RNA Mini Kit (Cat no. 12183018, Invitrogen, United Kingdom). First-strand cDNA was prepared using a Primer Script First Strand cDNA Synthesis Kit (Cat no. 6210A, TaKaRa, Otsu, Japan). Quantitative-PCR (qPCR) was performed by using a Fast Start Universal SYBR Green Master (ROX) (Cat no. 04913914001, Roche, Mannheim, Germany). The specific primers for qPCR are listed in **Supplementary Table S2**. *Cullin* and the rice *Ubiquitin* gene were chosen as references for the RT-qPCR in *S. italica* tissues and the rice transgenic lines, respectively, according to the previous reports (Martins et al., 2016; Wang Y. et al., 2017). The data were analyzed by using an Applied Biosystems 7300 Analyzer (Applied Biosystems, Foster City, CA, United States).

### Flow Cytometry

For flow cytometry, approximately 100 mg of proliferating first leaves were lacerated into 1 ml of cold nuclear isolation buffer (Lin et al., 2012) and then filtered with a 74 μm mesh. The suspension liquid was then stained with 2.5 mg ml^-1^ of 4′,6-diamidino-2-phenylindole (DAPI) for 10 min and analyzed on a MoFlo XDP cytometer (Beckman Coulter, CA, United States). For each test, 8000 nuclei were record to detect the ploidy level.

### Yeast One-Hybrid Analysis

For the yeast one-hybrid analysis, the bait sequences containing adaptors and three tandem repeats of binding site sequence are listed in **Supplementary Table S3** (EBS, mEBS). The coding sequences of prey proteins were amplified with the primers listed in **Supplementary Table S3** (for *Seita.4G108100*: primer pairs “4G100”; for *Seita.1G314900*: primer pairs “1G900”). The yeast one-hybrid assay was operated by using a Matchmaker Gold Yeast one-hybrid system (Cat no. 630491, Clontech, Mountain View, CA, United States).

### Transcriptome Sequencing Analysis

Yugu1 and *sistl2* plants were grown under the conditions of 16 h light and 8 h dark at 28°C for 4 weeks. The fourth extending leaves of *sistl2* and Yugu1 were harvested for total RNA extraction. The transcriptome sequencing was then performed and the cDNA libraries of *sistl2* and the WT were constructed in accordance with the Illumina sequencing manual and then sequenced on an Illumina HiSeq 2500 Genome Analyzer (Illumina, San Diego, CA, United States) with three independent biological replicates. Raw sequencing data were deposited with EMBL-EBI in the European Nucleotide Archive database under the accession number ERP106657. The qRT-PCR primers for the validation of the RNA-seq results are listed in **Supplementary Table S9**.

### Carbon Isotope Determination and Low CO_2_ Treatment

Approximately 50 mg of mature leaf samples at the heading stage of Yugu1 and *sistl2* were harvested. The samples were dried off at 80°C for 24 h. Pulverized samples were then disposed in accordance with a method described in a previous study (Cousins et al., 2008). The carbon isotopes were measured by using a Thermo Fisher Delta V isotope ratio mass spectrometer and calculated from a previously described equation (Stutz et al., 2014).

To verify how a low CO_2_ environment impacts *SiSTL2* and photosynthesis-associated genes, 4-week-old plants of the WT and *sistl2* were grown in a CO_2_ level control box. The CO_2_ level was set to 40 ppm with 16 h of light and 8 h of dark at 28°C. To avoid the influence due to the daily fluctuation of the genes, samples are harvested from the same parts of the leaves at the same time in each day (10 o’clock a.m.).

## Results

### Phenotypic Characterization of the *S. italica sistl2* Mutant

**FIGURE 1:**
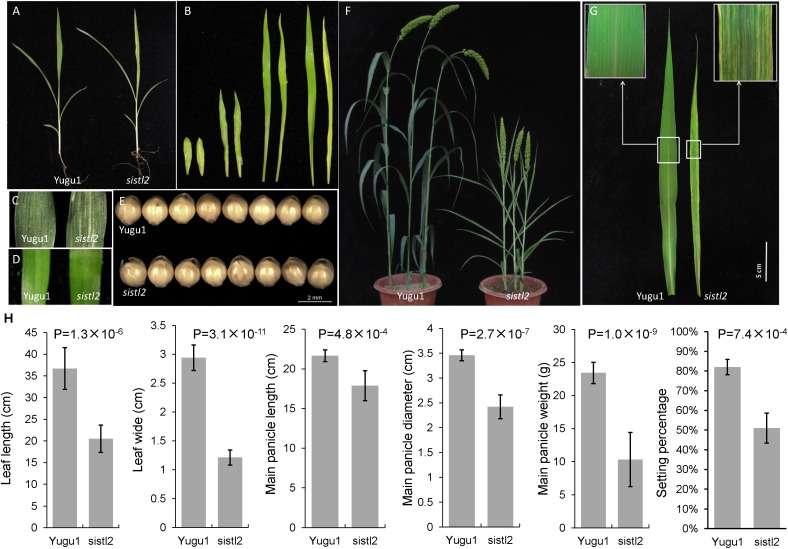
Phenotypic characterization of *sistl2* mutant. **(A)** The four-leaf stage seedlings of Yugu1 and *sistl2.* **(B)** The first four leaves of Yugu1 and *sistl2* were placed in order from left to right. In each couple, the left one is Yugu1, and the right one is *sistl2*. **(C)** The first leaf of Yugu1 and the mutant. **(D)** The third leaf of Yugu1 and the mutant. **(E)** The seeds of Yugu1 and *sistl2*. **(F)** Heading stage phenotypes of Yugu1 and *sistl2.* **(G)** The mature leaves of Yugu1 and *sistl2* of heading stage. **(H)** The leaf length and leaf width were clearly reduced in addition to the panicle size, panicle weight, and the seed setting percentage of Yugu1 and *sistl2.* The leaves used for size measurement are mature top second leaves of Yugu1 and *sistl2* at heading stage, respectively. The means and standard deviations were obtained from five independent leaf samples. Statistical analysis was performed with the *t*-test.

The *S. italica* stripe leaf mutant *sistl2* was isolated from EMS-induced Yugu1 cultivar. Before the three-leaf stage, the phenotype of the mutant was normal, except for the white striped first leaf (**Figures 1A–D**). From the fourth leaf, *sistl2* begins to display white striped leaves (**Figure 1B**). At the shooting and heading stage, *sistl2* exhibited decreased plant height, impaired plant development, and notable narrow leaves covered with white stripes (**Figures 1F,G**). However, the grain size of the mutant was not affected (**Figure 1E**).

Several key agronomic traits of WT and *sistl2* were also surveyed. The results proved that the entire plant development of *sistl2* was arrested (**Supplementary Table S4**). It is worth noting that the leaf length and leaf width were clearly reduced; in addition, the panicle size, panicle weight, and seed setting percentage in *sistl2* also decreased extensively (**Figure 1H** and **Supplementary Table S4**). These results indicated that leaf extension and reproductive development were impaired in *sistl2*.

### C_4_ Typical Plant Leaf Structure and Development Was Seriously Defective

**FIGURE 2:**
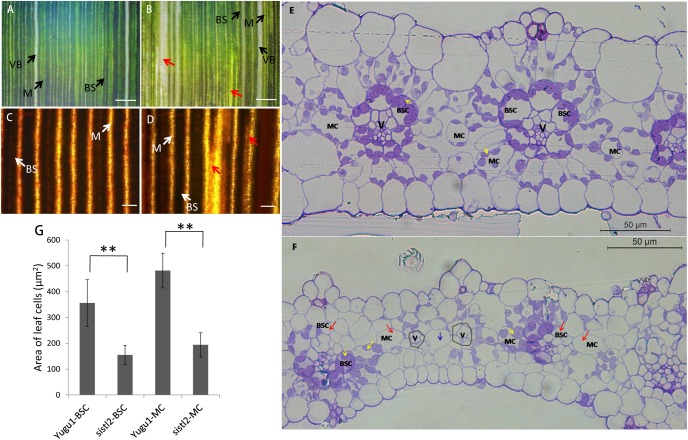
Leaf structure of Yugu1 and *sistl2*. **(A,B)** The leaves of Yugu1 and *sistl2* observed by portable digital microscope. BS, bundle sheath. M, mesophyll tissue. Red arrows show abnormal leaf veins. Bars: 500 μm. **(C,D)** The leaves of Yugu1 and *sistl2* dyed by I_2_–KI and viewed by using a light microscope. Red arrows show abnormal leaf veins. BS, bundle sheath; M, mesophyll tissue. Bars: 100 μm. **(E,F)** Leaf cross sections of Yugu1 and *sistl2*, respectively. Yellow arrows, chloroplasts in normal BSCs/MCs; Red arrows, BSCs/MCs contain no chloroplasts; Gray polygons show the leaf veins with undeveloped BSCs. Blue arrow, only one MC between two leaf veins (V). The type of the cell between two leaf veins was judged by the structure of its chloroplast as elucidated in **Supplementary Figure R1**. **(G)** The areas of BSCs and MCs in Yugu1 and *sistl2*. Twenty medium-sized cells from three horizons were calculated. The statistical analysis was performed by *t*-test. ^∗∗^*P* = 0.01.

To investigate the changes in the leaf anatomy structure in *sistl2*, we observed the leaves by using a portable digital microscope. The results showed that the leaf veins of WT were complete. Bundle sheath (BS, contain more and larger chloroplasts), mesophyll tissue (M, contain less and smaller chloroplasts), and vascular bundles (VBs, without any chloroplasts) array neatly (**Figure 2A**). In contrast, some leaf veins of *sistl2* are misshapen and array in a disordered fashion (**Figure 2B**, red arrows). This result can also be confirmed by the I_2_–KI staining on the leaves of WT and *sistl2* (**Figures 2C,D**). Because of different starch accumulation in chloroplast, BS and M can be dyed different color by I_2_–KI. The BS and M of WT were dyed brown and yellow, respectively, and arrayed neatly. These results showed that the leaf veins of WT were intact and regular (**Figure 2C**). However, some veins of *sistl2* are discontinuous (**Figure 2D**, red arrows).

To further assess the anatomical traits, cross sections of the leaf were obtained (**Figures 2E,F**). In the leaves of Yugu1, the Kranz structure is regular; mesophyll cells (MCs) and bundle sheath cells (BSCs) contain many chloroplasts which are dyed purple (**Figure 2E**, yellow arrows). As a typical C_4_ plant, *S. italica* has a constant leaf vein distance; between the two leaf veins (V) there are always two to three MCs in WT leaves (**Figure 2E**). Compared with the WT, the Kranz structure of *sistl2* was irregular. Although some BSCs and MCs are normal (**Figure 2F**, yellow arrows), many have fewer chloroplasts or even do not contain any chloroplast (**Figure 2F**, red arrows). In addition, for some leaf veins of *sistl2*, the BSCs appear to be undeveloped or absent (**Figure 2F**, gray polygons) and there is only one MC between the two veins (**Figure 2F**, blue arrow). In addition, it is worth noting that the sizes of BSCs and MCs in *sistl2* were decreased to 43.52 and 40.27% of that in the WT, respectively (**Figure 2G**). These results indicated that the chloroplast biogenesis and the leaf cell expansion of *sistl2* were inhibited and that the leaf anatomical structure was also impaired.

**FIGURE 3:**
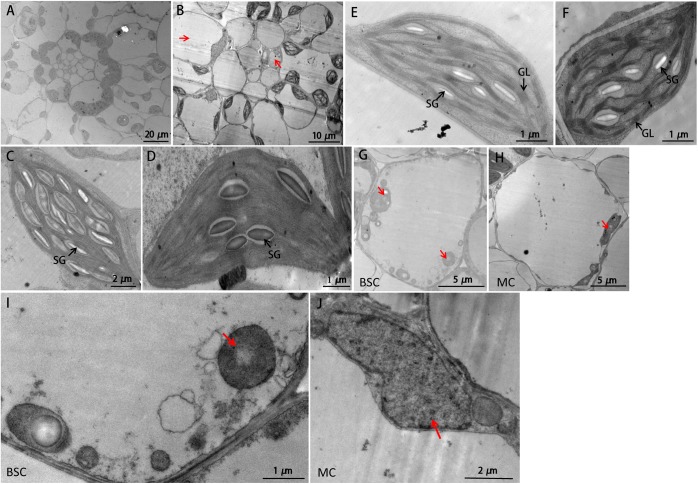
TEM images of WT and *sistl2*. **(A,B)** Kranz structures of Yugu1 and *sistl2*, respectively. Red arrows, abnormal BSCs and MCs. **(C,E)** The chloroplasts in BSC and MC of Yugu1, respectively. The structure of an individual BSC chloroplast (WT) was shown in **Supplementary Figure R2**. **(D,F)** The chloroplasts in normal BSC and MC of *sistl2*, respectively. SG, starch granules; GL, grana lamella. Abnormal BSC **(G)** and MC **(H)** in *sistl2*. Red arrows show the undeveloped chloroplasts. **(I,J)** Undeveloped chloroplast in BSC and MC.

To further investigate the influences on *sistl2* chloroplast biogenesis, mature leaves of *sistl2* and WT were observed by TEM, as shown in **Figure 3**. The Kranz structures of the WT and the *sistl2* mutants are shown in **Figures 3A,B**, respectively. Normal and abnormal (red arrows in **Figure 3B**, these cells have less or no chloroplast) BSCs/MCs could be observed in *sistl2*. The chloroplasts in the normal MCs of *sistl2* were similar to the WT, containing fully developed stroma lamellas and granum lamellas (**Figures 3E,F**). As *S. italica* is an NADP-ME type C_4_ plant, the BSCs contain only stroma lamellas. The chloroplasts in normal BSCs of *sistl2* also have well-developed stroma lamellas (**Figures 3C,D**). However, some BSC chloroplasts of *sistl2* contained fewer starch grains relative to the number found in Yugu1 (**Figures 3C,D**). In contrast, the chloroplasts in the abnormal BSCs/MCs of *sistl2* seem to be undeveloped (**Figures 3G,H**; red arrows). These results further clarify that the formation of Kranz structure, the leaf cell development, and chloroplast biogenesis in *sistl2* were significantly impaired.

### Genetic Mapping of the *SiSTL2* Locus and Bioinformatics Analysis

**FIGURE 4:**
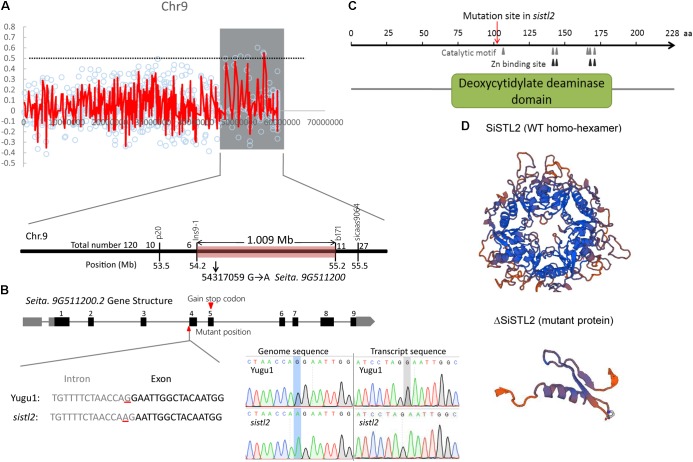
**(A)** Genetic mapping of the *SiSTL2* locus. In top panel, Y-axis means the ΔSNP index, X-axis means physical location. Red lines show the ΔSNP indexes of different SNPs on chromosome. Gray box shows the candidate region obtained by MutMap+. The bottom panel indicates that the gene is narrowed to a 1.009 Mb region between Ins9-1 and b171 by 120 F_2_ individuals. **(B)** The top of the figure shows the gene structure. Black boxes, exons; gray boxes, UTRs; gray lines, introns. The bottom of the figure shows the transcript variation proved by sequencing. Left bottom: gray text, intron; black text, exon; red lines, mutant position. Right bottom: the blue boxes and gray box show the mutant positions in genome sequence and transcript sequence, respectively. **(C)** The conserved functional domain and motifs of the SiSTL2 protein. Gray triangles show the six catalytic motifs, black triangles show the four Zn binding sites, and red arrows show the mutant position. **(D)** 3D protein structures of SiSTL2 (WT homo-hexamer) and ΔSiSTL2 (mutant protein).

Fine mapping was performed by the means of MutMap+ by using the M_4_ population (Fekih et al., 2013). The *SiSTL2* locus was mapped to a 12 Mb genomic region from 46.8 to 58.9 Mb on chromosome 9 (**Figure 4A**, gray box). In accordance with the requirement for the index of homozygous recessive mutation should be greater than 0.9, four putative mutation sites were identified (**Supplementary Table S5**). In addition, an F_2_ mapping population was generated from a cross between *sistl2* with the SSR41 cultivar. Through the use of this F_2_ population (120 individuals with striped leaves), a 1 Mb genomic region between the In-Del marker Ins9-1 and the SSR marker b171 was defined (**Figure 4A**). Among those four putative mutations, only the mutant site of a G to A mutation at 54,317,059 on chromosome 9 is included in this region and it is predicted to lead a splice site alteration at *Seita.9G511200*. *Seita.9G511200* was assumed to have nine exons and eight introns, with the G to A mutation located at the last base of the third intron. The transcript sequence verified that this mutation caused the first base of the fourth exon to be altered to the last base of the upstream intron, which further leads to a single-base deletion of the coding sequence (**Figure 4B**) and resulted in a frame shift and a premature stop codon at the fifth exon.

Gene annotation revealed that *SiSTL2* encoded a putative *Setaria* deoxycytidylate (dCMP) deaminase. *SiSTL2* encoded a 228 amino acid peptide chain. The protein structure domain, as predicted by NCBI, showed that SiSTL2 comprised a dCMP deaminase (DCD) domain from amino acids 71 to 184. Six catalytic motifs and four Zn^2+^ binding sites were located in this domain, which were essential for catalytic function. However, the mutation occurred at amino acid 107. As a result, the mutant SiSTL2 protein (ΔSiSTL2) lacks all the functional motifs and a large section of the DCD domain, which caused a defective protein function (**Figure 4C**). The SiSTL2 3D protein structure of the WT and *sistl2* were modeled (**Figure 4D**). These results showed that the SiSTL2 protein contained five alpha helixes and eight beta sheets, which functioned as a homo-hexamer, which forms a hexagon. However, ΔSiSTL2 loses four alpha helixes and six beta sheets, so that it cannot form any homo-multimer (**Figure 4D**).

### *SiSTL2* Can Complete the Phenotype of the *OsDCD* Mutant

**FIGURE 5:**
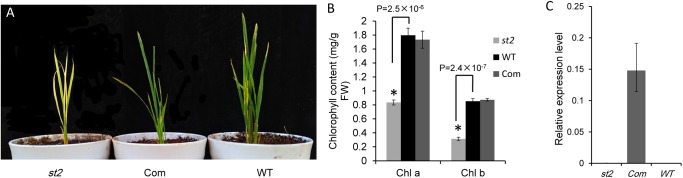
**(A)** Phenotypic comparison of *st2*, complemented T_0_ (Com), and the WT of *st2*. The *st2* mutant and WT were grown 28 days in the green house, and the completed T_0_ was 14 days after transplant. **(B)** The chlorophyll content of *st2*, Com, and WT. The means and standard deviations were obtained from three independent leaf samples. Statistical analysis was performed with the *t*-test. ^∗^*P* = 0.05. **(C)** The transcription levels of *SiSTL2* in *st2*, WT, and transgenic lines. The means and standard deviations are obtained from three independent leaf assays.

The protein sequence BLAST showed that SiSTL2 was a homologous protein of *Oryza sativa* OsDCD. Owing to the difficulty of *S. italica* transformation, we choose the *OsDCD* mutant *st2*, which has a similar phenotype to *sistl2*, to perform the complementation assay. A fragment harboring a 6.75 kb genome sequence of *Seita.9G511200*, which contains a promoter and a 3′-UTR is introduced into *st2*. All five positive transgenic lines exhibited normal phenotypes. The phenotype and chlorophyll content of complemented T_0_ (Com) was restored to WT (**Figures 5A,B**). The transcript of *SiSTL2* accumulated abundantly in transgenic lines, but did not exist in *st2* and the WT (**Figure 5C**). These results indicated that SiSTL2 had a similar function to OsDCD and was responsible for the stripe-leaf phenotype.

### DCD Deamination Activity and Chloroplast Development Are Impaired in *sistl2*

**FIGURE 6:**
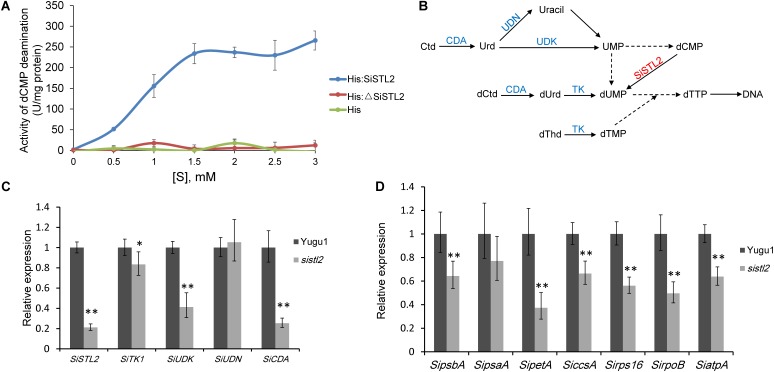
**(A)** Enzyme activity assay of SiSTL2. One unit of activity refers to deamination of 1 μM dCMP per minute under the conditions of the assay. The activity of SiSTL2 was determined with increasing concentrations of dCMP. The standard deviations are obtained from three independent assays. **(B)**The *de novo* biosynthesis pathway and salvage pathway of dTTP according to previous study (Leija et al., 2016) with several changes based on *phytozome* database. The DCD homolog SiSTL2 is shown in red and enzymes in salvage pathway are shown in blue. TK, thymidine kinase; UDK, uridine kinase; UDN, uridine nucleosidase; CDA, cytidine deaminase. The dotted lines indicate the abbreviated multi-steps. **(C)** The relative expression levels of *SiSTL2* and pyrimidine triphosphate salvage-associated genes **(D)** The relative expression levels of chloroplast development-associated genes. *SipsbA*, encodes PS II protein D1; *SipsaA*, encodes PS I P700 apoprotein A1; *SipetA*, encodes cytochrome f; *SiccsA*, encodes cytochrome c heme attachment protein; *Sirps16*, encodes cytochrome c heme attachment protein; *SirpoB*, encodes RNA polymerase beta subunit; *SiatpA*, encodes ATP synthase CF1 alpha subunit. The means and standard deviations were obtained from three independent samples. Statistical analysis was performed with *t*-test. ^∗^*P* = 0.05; ^∗∗^*P* = 0.01.

To gain whether SiSTL2, the homolog of OsDCD, has DCD deamination activity, wild-type SiSTL2 protein with histidine tag (His-SiSTL2) and mutant SiSTL2 protein with histidine tag (His-ΔSiSTL2) were purified (**Supplementary Figure S1**). Then an *in vitro* assay was performed to test the deamination activity of SiSTL2 and ΔSiSTL2 (**Figure 6A**). The kinetic assay showed that with the substrate concentration increased, the deamination activity of the recombinant His-SiSTL2 rose and reached maximum. However, His-ΔSiSTL2 almost has no deamination activity (**Figure 6A** and **Supplementary Figure S1**, histidine tag protein as a negative control). This result indicate that SiSTL2 has deamination activity and can catalyze the deamination of dCMP. However, when the C terminal of the protein is deleted, ΔSiSTL2 lost the deamination activity.

To study the influence on transcription level caused by the mutation of *SiSTL2*, the relative expression levels of *SiSTL2* were verified in leaves at the seedling stage (**Figure 6C**). The results showed that the transcription levels of *SiSTL2* in the mutant was significantly reduced compared with Yugu1. This result indicated genes involved in *de novo* pathway and the salvage pathway of dTTP biosynthesis were affected. As the pyrimidine triphosphate salvage pathway is also essential for dTTP synthesis, the relative expression levels of several salvage pathway genes (*SiTK1*, *SiUDK*, *SiUDN*, and *SiCDA*) were also determined (**Figures 6B,C**). The results showed that the expression of salvage pathway genes in the mutant was also decreased. These results suggested that the mutation in *SiSTL2* influenced both the *de novo* pathway and the salvage pathway of dTTP biosynthesis.

To explain the white stripe formation in *sistl2*, the expression levels of chloroplast development associated genes were also detected. The results indicated that the transcription levels of most of these genes were significantly lower in *sistl2* (**Figure 6D**), which suggested that the chloroplast development was impaired at the transcriptome level. However, whether this transcription decline is result from the dTTP biosynthesis is still unclear.

### *SiSTL2* May Be Controlled by E2F and Is Involved in Cell Cycle Regulation

**FIGURE 7:**
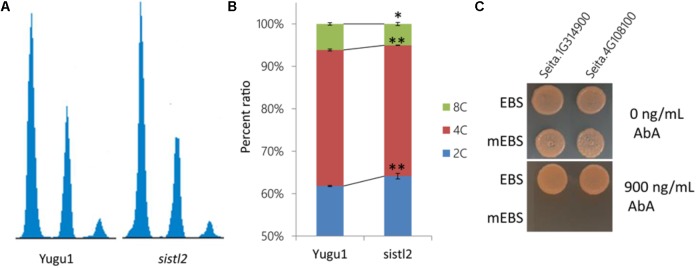
**(A)** Ploidy distribution in the proliferating leaves of Yugu1 and *sistl2*. **(B)** Percentage ratios of 2C, 4C, and 8C cells in Yugu1 and *sistl2*. The means and standard deviations were obtained from three independent samples. ^∗^*P* = 0.05; ^∗∗^*P* = 0.01. **(C)** The interactions between the *SiSTL2* promoter and two E2F family members. EBS means the E2F binding site in SiSTL2 promoter. mEBS is a random sequence used as a negative control. AbA, *Aureobasidin A*. 900 ng/ml is the lowest concentration of AbA that can avoid self-activating effects; 0 ng/ml AbA is the negative control.

Deoxycytidine monophosphate deaminase decreases can sometimes impair the mitosis cell cycle progression (Niu et al., 2017). Thus, the DNA ploidy levels of leaf cells in the WT and *sistl2* were determined by using flow cytometric analysis. The first leaves of Yugu1 and *sistl2* were harvested at 48 h after germination sprout. The results displayed that *sistl2* has higher percentage of 2C cells, but less 4C and 8C cells than Yugu1 (**Figures 7A,B**). In other words, in *sistl2*, more cells were present in the G1/S-phase and fewer cells were in the G2/M-phase than those in the WT. This result indicated that the mitotic cell cycle of *sistl2* was delayed at the transition from the G1/S- to the G2/M- phase.

It has been reported that the expressions of DCD and many other nucleotide biosynthetic genes were coordinated with cell cycle progression and regulated by some cell cycle-associated enzymes (Ke et al., 2005). We also considered whether the expression of *SiSTL2* was controlled by any cell cycle-related proteins. Thus, the *cis*-acting elements in the *SiSTL2* promoter were predicted and an E2F transcription factor binding site (EBS) (CCCCAAAGTTTCCCGCGCTTA) was found at -99 to -119 bp upstream of the initiation codon of *SiSTL2*. A random sequence (mEBS) (TCGCATCTGCCACCTCAGTAC) of the same length as EBS was considered to represent a negative control. Then, we verified the interactions between the EBS element and the SiE2F proteins by using the yeast one-hybrid assay (**Figure 7C**). The results showed that *Seita.4G108100* and *Seita.1G314900*, which are homologs of E2Fe, could bind to the EBS of the *SiSTL2* promoter and activate the expression of the *Aureobasidin A* (AbA) resistance gene in yeast strains. E2F family members are TFs that are closely related to the regulation of cell cycle progression. Collectively, these results suggested that *SiSTL2* may be regulated by cell cycle-related E2Fe and impacted cell cycle progression. Defective *SiSTL2* resulted in blockage of the cell cycle.

### Comparison of Genome-Wide Transcriptomes of WT and *sistl2*

Deoxycytidine monophosphate deaminase is a key enzyme in the regulation of the synthesis of dTTP. The lack of dTTP may impact DNA replication and further influence the other aspects of growth and development. The *sistl2* mutant showed comprehensive abnormal phenotypes of plant height, panicle size, seed-set rate, and especially leaf development. Thus, to reveal the signaling pathways and the regulatory networks that changed in the *sistl2* mutant, and to explain the relationships between SiSTL2 and leaf or chloroplast development, the genome-wide transcriptomes of leaves of Yugu1 and *sistl2* were compared. The fourth leaves during extension were chosen as the material for RNA sequencing.

**FIGURE 8:**
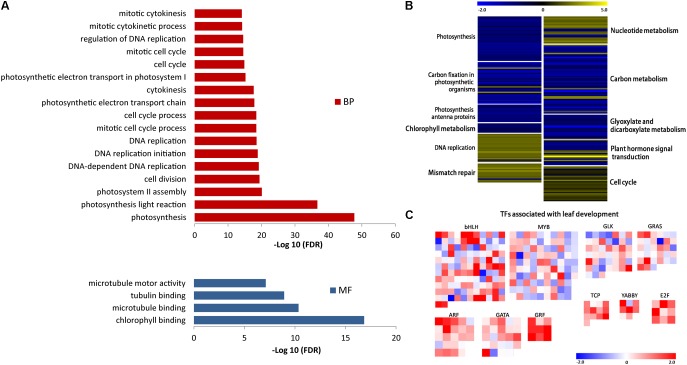
**(A)** DEGs with enriched GO terms. BP, biological process; MF, molecular function. **(B)** Expression analysis of DEGs in KEGG pathways associated with mutant phenotype regulation. Each group of color-block represent a KEGG pathway. The pathways name are shown beside each block group. Color of each block represent the value of log_2_(RPKM-mutant/RPKM-WT), reflecting the expression pattern of each gene in the mutant compared with WT. Expression patterns are shown as the color bar ranging from –2.0 to 5.0. **(C)** Transcript abundance differences of DEGs in TFs families responsible for C_4_ photosynthesis and leaf development. Color of each block represent the value of log_2_(RPKM-mutant/RPKM-WT), reflecting the expression pattern of each TF encoding gene in the mutant compared with WT. Expression patterns are shown as the color bar ranging from –2.0 to 2.0. Data are obtained from three independent biological replicates.

The analysis detected 2694 different expressed genes (DEGs) between Yugu1 and *sistl2* (**Supplementary Table S6**). The enriched KEGG pathways of the top 10 DEGs are listed in **Table 1**. The data showed that the DEGs were most concentrated in photosynthesis associated pathways (photosynthesis, carbon fixation in photosynthetic organisms, and photosynthesis-antenna proteins) and DNA replication associated pathways (DNA replication and mismatch repair). Genes associated with carbon metabolism and secondary metabolite (fatty, glyoxylate, and phenylpropanoid) biosynthesis and metabolic pathways were also extensively changed. The results of Gene Ontology (GO) analysis showed that DEGs were enriched in GO terms related to photosynthesis, cell cycle progression, DNA replication, and microtubule (involved in spindle formation) (**Figure 8A**).

**Table 1:** DEGs most enriched KEGG pathways.

Pathway	DEGs with pathway	All genes with pathway	*P*-value	*Q*-value	Pathway ID
	annotation (454)	annotation (5291)			
Photosynthesis	33 (7.27%)	82 (1.54%)	2.97 × 10^-15^	3.09 × 10^-13^	ko00195
Carbon fixation in photosynthetic organisms	31 (6.83%)	83 (1.56%)	2.40 × 10^-13^	1.25 × 10^-11^	ko00710
Photosynthesis – antenna proteins	13 (2.86%)	16 (0.3%)	4.69 × 10^-12^	1.63 × 10^-10^	ko00196
DNA replication	21 (4.63%)	67 (1.27%)	7.04 × 10^-8^	1.83 × 10^-6^	ko03030
Carbon metabolism	51 (11.23%)	293 (5.5%)	3.70 × 10^-7^	0.000007	ko01200
Fatty acid elongation	15 (3.3%)	48 (0.91%)	5.64 × 10^-6^	0.000106	ko00062
Mismatch repair	14 (3.08%)	49 (0.93%)	0.000039	0.000581	ko03430
Glyoxylate and dicarboxylate metabolism	18 (3.96%)	77 (1.46%)	0.000063	0.000815	ko00630
Phenylpropanoid biosynthesis	41 (9.03%)	264 (4.99%)	0.000106	0.001226	ko00940
Phenylalanine metabolism	12 (2.64%)	50 (0.95%)	0.000815	0.008471	ko00360


*The English in this document has been checked and edited by two professional editors, both native speakers of English. For a certificate, please see the upload file “Certificate_of_editing-SRATA_1.pdf.”*

Given the function of SiSTL2 and the phenotypes of the *sistl2* mutant, we selected DEGs in the pathways associated with photosynthesis, DNA replication, chlorophyll metabolism, hormone signals, and carbon metabolism and analyzed the changes in expression (**Figure 8B** and **Supplementary Table S7**). Most of the DEGs in photosynthesis-associated pathways and chlorophyll metabolism were down-regulated, which was consistent with the decreased capacity of photosynthesis and chlorophyll content of *sistl2*. Almost all of the DEGs in nucleotide metabolism, DNA replication and mismatch repair pathways were up-regulated; this may be caused by feedback regulation of the SiSTL2 functional defect. DEGs in carbon metabolism and glyoxylate/dicarboxylate metabolism generally were down-regulated, indicating that energy conversion may be impaired in *sistl2*. DEGs in hormone signal pathways showed great changes, especially several auxin and cytokinin response regulator genes were sharply up-regulated (*SiIAA26*, 1.80 fold; *SiLAX2*, 1.74 fold; *SiARR12*, 2.14 fold; *SiARF5*, 2.31 fold; *SiDFL1*, 5.50 fold; *SiARR9*, 1.99 fold; *SiTGA9*, 2.58 fold). This suggested that hormone regulators that promote growth respond positively to the development of plant retardation. Most DEGs that participate in cell cycle regulation were up-regulated, which was consistent with the results of flow cytometric analysis and the yeast one-hybrid assay.

The expression changes of some associated TFs are summarized in **Figure 8C** and **Supplementary Table S8**. It is noteworthy that some TFs families are generally up-regulated, such as Teosinte Branched 1/Cycloidea/Proliferating Cell Factor (TCP), E2F, Growth Regulating Factor (GRF), YABBY, and Auxin Response Factor (ARF). Among these TFs families, ARF, E2F, and GRF control the leaf growth and development by regulating cell proliferation (Kim et al., 2003;Horiguchi et al., 2005, 2006; Magyar et al., 2005; Guilfoyle and Hagen, 2007; Lim et al., 2010). TCP family members are responsible for the progress of leaf expansion and development (Martin-Trillo and Cubas, 2010; Efroni et al., 2013). YABBY can regulate the leaf axial development (Sarojam et al., 2010). Additionally, some members of ARF and GRF are speculated to be associated with C_4_ photosynthesis in maize (Wang et al., 2013; Ding et al., 2015). However, some other TFs families also may be associated with leaf patterning, plastid biogenesis, and photomorphogenesis, such as MYB, GLK, bHLH, GRAS, and GATA (Wang et al., 2013). These TFs are also speculated to participate in C_4_ photosynthesis (Wang et al., 2013; Wang P. et al., 2017). Members of these TFs families are numerous. In *sistl2* mutant, many members of these TFs up-regulated or down-regulated sharply compared with that in WT. This indicates that the expression of these TFs are influenced in the mutant (**Figure 8C**). Collectively, the leaf development and C_4_ photosynthesis may be impaired because of the mutation of *SiSTL2*. In addition, to validate the RNA-seq, 13 genes were selected and detected by qRT-PCR (**Supplementary Figure S2**). These genes are associated with photosynthesis, DNA replication, and cell cycle, encoding PS I/II reaction center proteins (Seita.J018400, Seita.3G149000, Seita.3G184900, and Seita.6G032200), chlorophyll binding protein (Seita.6G158500), DNA polymerase subunits (Seita.6G050100 and Seita.5G394500), DNA replication licensing factor (Seita.1G352100), cycle division control proteins (Seita.4G045900 and Seita.2G283000), and cyclins (Seita.9G171000, Seita.5G005800, and Seita.5G352700). The consequences are correspond to that in RNA-seq.

### *SiSTL2* Affects C_4_ Photosynthesis Capacity in Foxtail Millet

**FIGURE 9:**
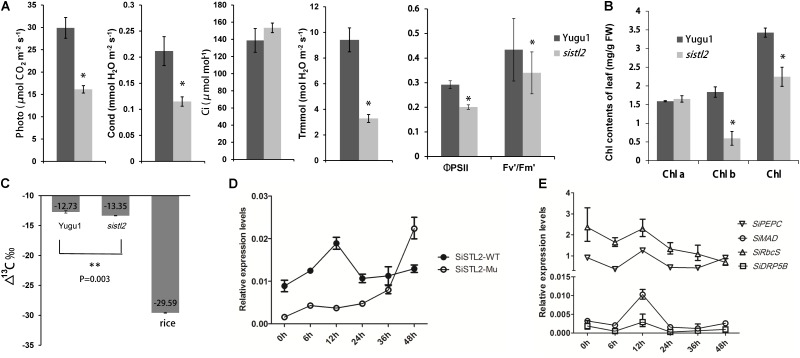
**(A)** Photosynthetic parameters in WT and *sistl2*. Photo, net photosynthetic rate. Cond, stomata conductance. Ci, intercellular CO_2_ concentration. Trmmol, transpiration rate. The means and standard deviations were obtained from five independent leaf samples. Statistical analysis was performed with the *t*-test. ^∗^*P* = 0.05. **(B)** The chlorophyll contents of Yugu1 and sistl2. The means and standard deviations were obtained from five independent leaf samples. Statistical analysis is performed with the *t*-test. ^∗^*P* = 0.05. **(C)** ^13^C contents in leaves of WT, *sistl2*, and rice. ^∗∗^*P* = 0.01. **(D)** The expression patterns of *SiSTL2* gene in Yugu1 and the mutant under low CO_2_ treatment. **(E)** Expression patterns of the C_4_ photosynthesis genes in low CO_2_ conditions. PEPC, phosphoenolpyruvate carboxylase; MAD, malate dehydrogenase; RbcS, rubisco small submit; DRP5B, a dynamin-related family protein involved in chloroplast division and development (Pyke and Leech, 1994). The means and standard deviations were obtained from three independent samples.

To investigate the influence of SiSTL2 on the capacity for C_4_ photosynthesis, the photosynthetic index, chlorophyll fluorescence kinetic parameters, and chlorophyll (Chl) accumulation were detected in *sistl2*. The results showed that compared with Yugu1, the net photosynthetic rate of *sistl2* was decreased by 46.1%; the stomata conductance was decreased by 45.6%; the transpiration rate declined by 65.2%, whereas the intercellular CO_2_ concentration showed no significant difference compared with that in Yugu1, concurrently. This suggested that the photosynthetic capacity of *sistl2* was impaired, which led to the accumulation of intercellular CO_2_. The ΦPSII reflects the light absorbed by PS II that is used in photochemical reactions. Fv′/Fm′ indicates the excitation energy capture efficiency, which is also reflective of the light portion used in photochemistry. The significant decrease in these two indices of *sistl2* indicated that light-use efficiency was influenced by the mutation (**Figure 9A**). Interestingly, Chl *a* content was not influenced, whereas the Chl *b* content and total Chl content was significantly decrease in the *sistl2* mutant (by approximately 67 and 18%, respectively; **Figure 9B**). It has been reported that the degradation of Chl b is prior to Chl a, and can translate to Chl a (Schelbert et al., 2009). This is consist with our result.

As described in **Figure 3B**, the leaf Kranz structure of the *sistl2* mutant is defective. The results of RNA-seq also suggested that the C_4_ photosynthesis of *sistl2* may be affected. To confirm this, the stable carbon isotopes of the *sistl2* mutant, Yugu1, and a rice *Japonica cultivar*, as a C_3_ control, were measured. The atmosphere contains a small amount of ^13^C. When C_3_ plants fix CO_2_, Rubisco prefers ^12^CO_2_. However, the C_4_ photosynthetic CO_2_ fixation enzyme PEPC does not tend to function on ^12^CO_2_. Rubisco of C_4_ plants only exists in BSCs which segregates rubisco from air. As a result, ^13^C accumulates to a greater extent in C_4_ plants than in C_3_ plants. It has been reported that C_3_ plants and C_4_ plants can be identified by the value of Δ^13^C aaa (C_3_: -32 to -23aaa; C_4_: -19 to -6aaa) (Bender, 1971). Our data show thatΔ^13^Caaa in *sistl2* was significantly decreased by 4.87% (**Figure 9C**), although the value was still within the range of C_4_. Then, we tested the expression of *SiSTL2* and certain C_4_ photosynthesis-related genes that respond to the stress of low CO_2_ environments. The results showed that in Yugu1, the expression level of *SiSTL2* was rapidly up-regulated until 12 h, decreased to the initial level, and remained stable. However, in *sistl2*, the transcription level remained at a low level until 24 h and started to increase after 36 h (**Figure 9D**). This suggested that the expression of *SiSTL2* can respond in a timely manner to the low CO_2_ level. The mutation in *SiSTL2* resulted in a delayed response to the stress of a low CO_2_ environment. The C_4_ photosynthesis-related genes (Ding et al., 2015; Huang et al., 2017) showed a similar expression pattern to *SiSTL2* in low CO_2_ conditions, which increased to a peak value at 12 h and then returned to the initial level (**Figure 9E**). In conclusion, we speculated that *SiSTL2* might indirectly regulated C_4_ photosynthesis.

To further verify the connection between *SiSTL2* and C_4_ photosynthesis, transcript levels of 102 previously reported *S. italica* C_4_ candidate genes (Ding et al., 2015) in WT and *sistl2* are summarized from our RNA-seq data (**Supplementary Table S10**). According to the expression patterns differences between C_3_ and C_4_ species, these genes are divided into three types. Type I including genes that have higher expression levels in C_4_ leaves compared with C_3_ leaves. Type II genes show different expression patterns between C_4_ and C_3_ species. Type III genes have orthologs in C_4_ species but absent in C_3_ species (Ding et al., 2015). Comparison result showed that most of these genes (89 of 102; Type I, 18 of 20; Type II, 59 of 65; Type III, 12 of 17) also changed their expression patterns in *sistl2* mutant (**Supplementary Table S10**). Moreover, expression of 25.5% of these genes decreased more than twofolds in *sistl2* compared with WT. According to the previous study (Ding et al., 2015), C_4_ candidate genes which are associated with C_4_ photosynthesis in leaves and C_4_ evolution are also summarized, respectively (**Supplementary Figure S3**, right panel and **Supplementary Table S10**). The result showed that 87.8% C_4_ photosynthesis associated genes (43 of 49) and 86.0% C_4_ evolution associated genes (37 of 43) are down-regulated in *sistl2*. These results further indicated that the mutant in *SiSTL2* has some linkage with C_4_ photosynthesis.

## Discussion

### Comparison of DCD Mutant Characteristics in Foxtail Millet and Rice

In *sistl2*, the plant height and chlorophyll contents were significantly decreased, the leaves have white stripes (**Figure 1**). Furthermore, some leaf cells in *sistl2* contain fewer chloroplasts or even have no chloroplast (**Figures 2E,F**). All these phenotypes were quite similar to the mutants of *OsDCD* (Xu et al., 2014; Niu et al., 2017). This suggested that the function of SiSTL2 is similar to that of OsDCD. What’s more, similar phenotypes also exist in the mutants of other dNTP synthesis associated genes and other species (Yoo et al., 2009; Qin et al., 2017). Thus we speculate that there are some correlations between dNTPs synthesis and leaf/chloroplast development, which need more experiments and evidences to prove it. In our study, the cell cycle progression of *sistl2* was impaired (**Figures 7A,B**), DEGs were enriched in the cell cycle GO term (**Figure 8A**) and most of the cell cycle-associated genes were up-regulated (**Figure 8B**). In the *alr* mutant, the cell cycle progression was also delayed (Niu et al., 2017). This indicated that SiSTL2 played a similar role in cell cycle regulation to OsDCD. In addition, the dNTP pools in *alr* were unbalanced. This caused further defects in DNA replication and repair (Niu et al., 2017). Our results show that SiSTL2 also has deamination activity similar with OsDCD (**Figure 6A**). In *sistl2*, the results of RNA-seq showed that abundant DEGs were enriched in the DNA replication and mismatch repair pathways (**Table 1**). In addition, most DEGs associated with nucleotide metabolism, DNA replication, and mismatch repair were up-regulated, which may be caused by feedback control (**Figure 8B**). These results suggested that similar to OsDCD, SiSTL2 can regulate DNA replication and reparation. In addition, the expression levels of chloroplast development-associated genes in *sistl2* were significantly decreased relative to Yugu1 (**Figure 6C**). The same results were also obtained in *alr* mutant (Niu et al., 2017), indicating that SiSTL2 disruption can impair chloroplast development. In summary, SiSTL2 has a similar function to OsDCD, and has effects on DNA replication and reparation, cell cycle progression, and chloroplast development.

### SiSTL2 Participates in the Regulation of Cell Division and Influences Cell Expansion

The leaves of the *sistl2* mutant were significantly smaller than that of Yugu1 (**Figure 1H** and **Supplementary Table S4**). The smaller sizes of organs usually from a reduction in cell number or cell size. These two parameters always depend on cell proliferation and cell expansion.

In our study, the ratios of 4C and 8C cells are significantly lower in *sistl2* compared with the level in Yugu1 (**Figures 7A,B**). We also found that in the vasculature of *sistl2*, the number of MCs and BSCs was reduced (**Figure 2F**). This indicated that the cell cycle progression in *sistl2* was delayed and that the cell proliferation may be defective. RNA-seq also showed that cell cycle-related genes (**Figure 8A**) and TFs (E2F; Heuvel and Dyson, 2008) suggesting that cell cycle of *sistl2* is influenced. Some reports indicated that many dNTP synthesis-associated genes were controlled by cell cycle regulating proteins (Ke et al., 2005). RNR, as an upstream gene of DCD and the S-phase check point, can active the cell cycle going into S-phase under the control of cell cycle regulating transcription factor E2F5 (Lincker et al., 2004). The promoter of *SiSTL2* also has binding sites of SiE2Fe (**Figure 7C**). Thus, we speculated that SiSTL2, similar to RNR, maybe be correlated with cell cycle regulation. However, SiE2Fe is predicted to have the opposite function relative to E2F5 in cell cycle regulation. Therefore, the role of SiSTL2 in the cell cycle required further exploration.

However, the leaf cell sizes of both BSCs and MCs in *sistl2* were also significantly decreased (**Figures 2F,G**), which indicated that the cell expansion of *sistl2* was also impacted. The RNA-seq data showed that TFs associated with cell expansion, such as TCP and GRF (Kim et al., 2003; Martin-Trillo and Cubas, 2010), were up-regulated. This suggested that the mutation in SiSTL2 also impaired cell expansion. In addition, *sistl2* the decreases in cell sizes (by 43.52% in BSCs and 40.27% in MCs) were as strong as those in leaf length (44.1% reduction) and leaf width (58.8% reduction) relative to Yugu1 (**Figure 2G** and **Supplementary Table S4**). However, the cell cycle progression changes were not so strong (**Figure 7B**). Therefore, we surmised that SiSTL2 may participate in cell cycle regulation and influence the cell expansion. In *sistl2*, the smaller leaf size was mainly caused by defective cell expansion, with reduced cell proliferation a subordinate reason for the smaller leaf size.

### SiSTL2 May Have an Impact on C_4_ Photosynthesis

Chloroplast development is important for photosynthesis, and the Kranz leaf structure is a characteristic and precondition of C_4_ photosynthesis. In *sistl2*, some BSCs and MCs have fewer or even no chloroplasts (**Figures 2E,F**) and the expression of chloroplast development genes are also decreased (**Figure 6D**). This suggested that the mutation in *SiSTL2* may affect the chloroplast development at transcription level. Considering that the chloroplast genes transcription depend on both nucleus-encoded RNA polymerase and plastid-encoded RNA polymerase, we speculate that the defect of SiSTL2 may have some influence on the nucleus or plastid transcription system. Additionally, *sistl2* displayed some abnormal phenotypes with respect to Kranz structure, such as undeveloped veins, reduced BSCs and decreased mesophyll cell number between vascular bundles (**Figures 2E,F**). This may be the result of the restraints in both cell expansion and cell division. However, many members of the TFs families that regulate leaf development and leaf cell differentiation, such as YABBY, bHLH, GRAS, and GATA (Siegfried et al., 1999; Wang et al., 2013) are differently expressed in *sistl2*. These suggested that mutations in *SiSTL2* influenced the leaf development and leaf structure. Meanwhile, most genes in the carbon fixation pathways in photosynthetic organisms were down-regulated (**Figure 8A**); the expression of TFs may regulate C_4_ photosynthesis (ARF, MYB, and GLK) (Ding et al., 2015) changed extensively (**Figure 8B**). And most of the C_4_ candidate genes were down-regulated after *SiSTL2* was defected (**Supplementary Figure S3**). These suggest that there are some linkage between C_4_ photosynthesis and the mutation of *SiSTL2*. The ^13^C accumulation in *sistl2* was also significantly reduced (**Figure 9C**). This suggested that the C_4_ photosynthesis of the mutant was affected. Moreover, under the stress of a low CO_2_ environment, *SiSTL2* can respond to the up-regulation in the similar pattern compared with C_4_ photosynthesis genes (**Figures 9D,E**). Given that the regulation of dTTP synthesis by DCD is a relatively upstream biological pathway, we speculate that the effect of the *SiSTL2* mutation is global in plants, indirectly regulating the chloroplast development and C_4_ photosynthesis. As the decrease in chloroplast biogenesis, the abilities of both photosynthesis and carbon fixation were impaired seriously. In the conditions of low CO_2_, the mobilization of C_4_ photosynthesis genes requires sufficient dNTPs to ensure that the expression levels of *SiSTL2* and C_4_ photosynthesis genes are subsequently altered.

## Author Contributions

XD conceived the project. SZ and ST performed the data analysis and wrote the manuscript. SZ, CT, and ML conducted the experimental work. HZ provided the materials and performed the field trials. XD, ST, and GJ guided the experimental work. All authors read and approved the final manuscript.

## Conflict of Interest Statement

The authors declare that the research was conducted in the absence of any commercial or financial relationships that could be construed as a potential conflict of interest.

## Footnotes

1 https://phytozome.jgi.doe.gov/pz/portal.html

2 https://www.ncbi.nlm.nih.gov/Structure/cdd/wrpsb.cgi

3 http://www.rcsb.org/

4 http://plantregmap.cbi.pku.edu.cn/binding_site_prediction.php

## Supplementary Material

FIGURE S1 Western blot of recombinant proteins His-SiSTL2, His-ΔSiSTL2 and His tag expressed in *E. coli*. Marker shows molecular mass in kilodaltons.

FIGURE S2 The transcript levels of genes for validation of RNA-seq result.

FIGURE S3 The expression analysis of the genes associated with C_4_ photosynthesis in leaves and C_4_ evolution. Color of each block represent the value of log_2_(RPKM-mutant/RPKM-WT), reflecting the expression pattern of each gene in the mutant compared with WT. Expression patterns are shown as the color bar ranging from -2.0 to 2.0.

TABLE S1 SSR and In-Del markers for fine mapping.

TABLE S2 Primers used for qRT-PCR.

TABLE S3 Primers used for yeast one-hybrid.

TABLE S4 Characterization of agronomic traits of *sistl2* mutant.

TABLE S5 Putative mutation sites information.

TABLE S6 Genes that were differentially expressed in *sistl2* compared with the wild-type.

TABLE S7 DEGs in mutant phenotype-associated pathways.

TABLE S8 DEGs in phenotype-associated TFs families.

TABLE S9 The qRT-PCR primers for the validation of RNA-seq result.

TABLE S10 Transcript levels of C_4_ candidate genes.

FIGURE R1 Top panel, the red cycles show two leaf veins of *sistl2*, the red box shows the cell between two leaf veins. The bottom panel shows the chloroplast (red arrow) of the cell in the red box in the top panel. The granum lamellas can be observed clearly in the chloroplast.

FIGURE R2 Kranz structure of Yugu1, the *S. italica* wild type. A chloroplast of BSC was pointed out by a blue cycle and magnified in **(B)**. **(B)** The red arrows show the structures like grana in BSC chloroplast.
