# Supplementary figures and images for: SiSTL2 Is Required for Cell Cycle, Leaf Organ Development, Chloroplast Biogenesis, and Has Effects on C4 Photosynthesis in Setaria italica (L.) P. Beauv
Source: Front Plant Sci. 2018 Jul 30;9:1103. doi: 10.3389/fpls.2018.01103 (PMC6077218; doi:10.3389/fpls.2018.01103)

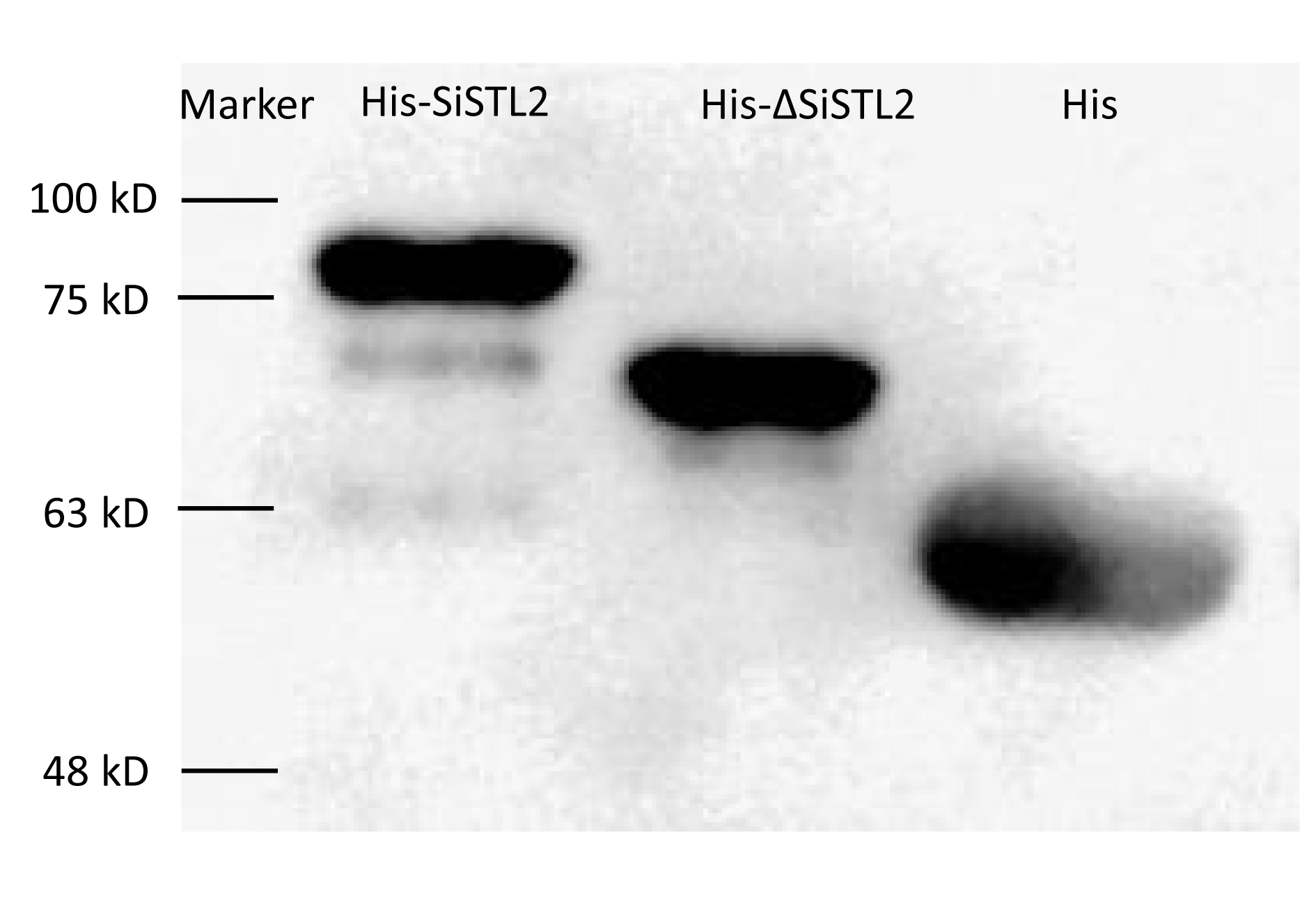

Supplement: FIGURE S1 [file Image_1.TIF]

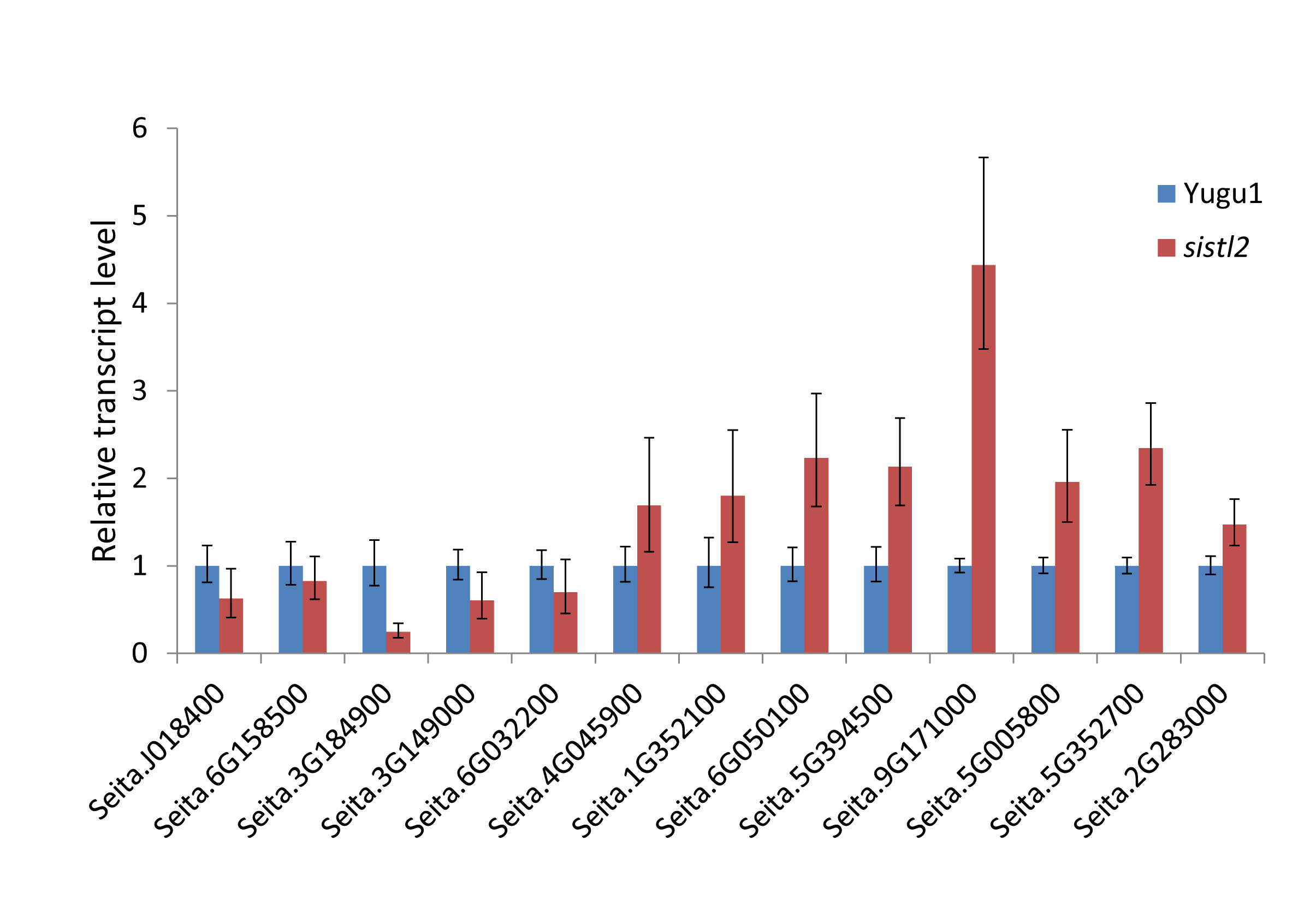

Supplement: FIGURE S2 [file Image_2.TIF]

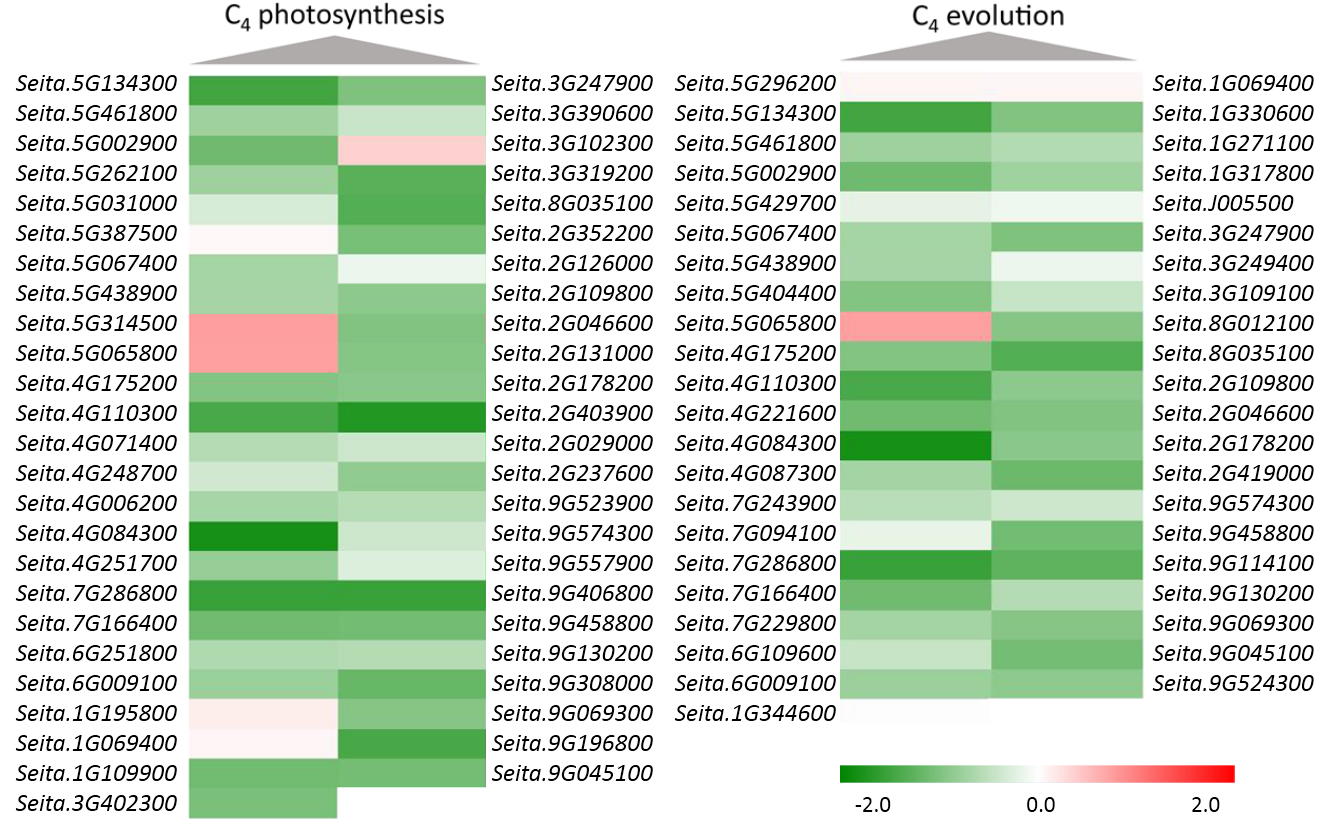

Supplement: FIGURE S3 [file Image_3.TIF]
