# Supplementary material for: SiSTL2 Is Required for Cell Cycle, Leaf Organ Development, Chloroplast Biogenesis, and Has Effects on C4 Photosynthesis in Setaria italica (L.) P. Beauv
Source: Front Plant Sci. 2018 Jul 30;9:1103. doi: 10.3389/fpls.2018.01103 (PMC6077218; doi:10.3389/fpls.2018.01103)
Supplement: TABLE S1 [file Table_1.DOC]

**Supplementary Table S1. SSR and In-Del markers for fine mapping**

| Primer name | Forward primer sequence | Reverse primer sequence |
| --- | --- | --- |
| P20 | ACCCTGGCATCTTCTT | CTTTACCCTCGGCTTG |
| Ins9-1 | AAACCCTTTGTGGAGT | TACCCGTTATTTGACC |
| b171 | AGCCATCGTTGTCTTCC | CCCGTATCCTCTTTCG |
| sicaas9064 | ATTCTACGGATGAAACAGC | AACAACACCGACAAGATAA |
