# Supplementary material for: SiSTL2 Is Required for Cell Cycle, Leaf Organ Development, Chloroplast Biogenesis, and Has Effects on C4 Photosynthesis in Setaria italica (L.) P. Beauv
Source: Front Plant Sci. 2018 Jul 30;9:1103. doi: 10.3389/fpls.2018.01103 (PMC6077218; doi:10.3389/fpls.2018.01103)
Supplement: TABLE S2 [file Table_2.DOC]

**Supplementary Table S2. Primers for qPCR**

| Primer name | Forward primer sequence | Reverse primer sequence |
| --- | --- | --- |
| *SiSTL2* | CCTTCAGGGAGCTGTTGTAA | AGGATCAAGCCATAGGACAC |
| *SiTK1* | CAAATCTCGAACCCCAAATC | TTAGGTGAGATTGGACTGGA |
| *SiUDK* | ACCACTGAATGCTGATAGTC | CTCTGTTCCCTTTGGCTAGT |
| *SiUDN* | ACACACTAGCAGTAAGCAAC | GAATGTCTGATTGACCAGTG |
| *SiCDA* | ATCATTGCGATGGTGGC | GTTGTACACGTGGAGACT |
| *SipsbA* | TGCTCACAACTTCCCTC | CCTACTCGGCATTTCAC |
| *SiatpA* | GTATGACCGCTTTACCAAT | GGAAACAGAAATACCCACA |
| *SipsaA* | AAGGCCATAAGGGTCTCTAT | ACCGACTATTAGAAATCCGC |
| *SipetA* | TATATGTAGGGGGAAACCGA | TTCCCCCTCTGAAACTAGAA |
| *SiccsA* | TCGTGTCATTAGTCTAGGGT | GTCCAGGTAATAAATGCCCA |
| *Sirps16* | ACCCTAGATTCTTTCCCTTG | GTTCTATTTGTCCCGAACCT |
| *SiropB* | GAGTCACTATGGGCGTATTT | GGACAAAGAATTTCCTGCTG |
| *SiPEPC* | CTTGGTTATGTGAGGGACAA | CAGTCACACAGGGATTTGTA |
| *SiMAD* | CTAATCTCCTCCACTCCCT | GTAAGCTGTTGAGAGACCC |
| *SiRbcS* | AGCTCCGGCTATGATATGTA | AAGGACAGGTATTGTAAGCC |
|  |  |  |
