# Supplementary material for: SiSTL2 Is Required for Cell Cycle, Leaf Organ Development, Chloroplast Biogenesis, and Has Effects on C4 Photosynthesis in Setaria italica (L.) P. Beauv
Source: Front Plant Sci. 2018 Jul 30;9:1103. doi: 10.3389/fpls.2018.01103 (PMC6077218; doi:10.3389/fpls.2018.01103)
Supplement: TABLE S3 [file Table_3.DOC]

**Supplementary Table S3. Primers used for yeast one-hebrid**

| Primer name | Forward primer sequence | Reverse primer sequence |
| --- | --- | --- |
| EBS | ATGAATTGAAAAGCTTCCCCAAAGTTTCCCGCGCTTACCCCAAAGTTTCCCGCGCTTACCCCAAAGTTTCCCGCGCTTAGAGCACATGCCTCGAG | CTCGAGGCATGTGCTCTAAGCGCGGGAAACTTTGGGGTAAGCGCGGGAAACTTTGGGGTAAGCGCGGGAAACTTTGGGGAAGCTTTTCAATTCAT |
| mEBS | ATGAATTGAAAAGCTTTCGCATCTGCCACCTCAGTACTCGCATCTGCCACCTCAGTACTCGCATCTGCCACCTCAGTACAGAGCACATGCCTCGAG | CTCGAGGCATGTGCTCGTACTGAGGTGGCAGATGCGAGTACTGAGGTGGCAGATGCGAGTACTGAGGTGGCAGATGCGAAAGCTTTTCAATTCAT |
| 4G100 | CAGATTACGCTCATATGATGGACGCCGCCTCCGCA | TCATCTGCAGCTCGAGATGATGATGTTTCCCGTGT |
| 1G900 | CAGATTACGCTCATATGATGTTGATGGCGATGGCGG | TCATCTGCAGCTCGAGTGTCTTGGTTCTGGTACTGT |
