# Supplementary material for: SiSTL2 Is Required for Cell Cycle, Leaf Organ Development, Chloroplast Biogenesis, and Has Effects on C4 Photosynthesis in Setaria italica (L.) P. Beauv
Source: Front Plant Sci. 2018 Jul 30;9:1103. doi: 10.3389/fpls.2018.01103 (PMC6077218; doi:10.3389/fpls.2018.01103)
Supplement: TABLE S4 [file Table_4.DOC]

**Supplementary Table S4. Characterization of agronomic traits** o**f** ***sistl2* mutant**.

| Trait |  | *yu-1* | *sistl2* | Change rate (%) | P value |
| --- | --- | --- | --- | --- | --- |
| Plant Height(cm) | ****** | 124.2±5.67 | 81.8±4.92 | ↓34.1 | <<0.01 |
| Leaf Length(cm) | ****** | 36.7±4.83 | 20.5±3.15 | ↓44.1 | <<0.01 |
| Leaf Wide(cm) | ****** | 2.94±0.22 | 1.21±0.13 | ↓58.8 | <<0.01 |
| Peduncle Length(cm) | ****** | 23.38±2.42 | 14.43±2.87 | ↓38.3 | <<0.01 |
| Main Panicle Length(cm) | ****** | 21.66±0.74 | 17.88±1.89 | ↓17.5 | 0.0005 |
| Main Panicle Diameter(cm) | ****** | 3.46±0.11 | 2.42±0.24 | ↓30.0 | <<0.01 |
| Panicle Branch Number(per 5 cm) | ****** | 20.60±1.67 | 30.57±3.51 | ↑48.4 | <<0.01 |
| Main Panicle Weight(g) | ****** | 23.42±1.60 | 10.31±4.10 | ↓56.0 | <<0.01 |
| Thousand Seeds Weight(g) |  | 3.01±0.36 | 2.91±0.12 | - | 0.299 |
| Seed setting percentage | ****** | 0.82±0.039 | 0.51±0.076 | ↓37.8 | 0.0007 |

**Means and standard deviations are obtained from ten independent plants. **** **Significantly different at P = 0.01. Arrows show the variation trends.**
