# Supplementary material for: SiSTL2 Is Required for Cell Cycle, Leaf Organ Development, Chloroplast Biogenesis, and Has Effects on C4 Photosynthesis in Setaria italica (L.) P. Beauv
Source: Front Plant Sci. 2018 Jul 30;9:1103. doi: 10.3389/fpls.2018.01103 (PMC6077218; doi:10.3389/fpls.2018.01103)
Supplement: TABLE S5 [file Table_5.DOC]

**Supplementary Table S5.Putative mutation sites information**

| **POS.** | **Mutant index** | **WT index** | **REF** | **ALT** | **Effect** |
| --- | --- | --- | --- | --- | --- |
| **Chr9:50815621** | 0.976 | 0.524 | T | A | Intergenic |
| **Chr9:51776997** | 1 | 0.613 | C | T | Synonymous coding |
| **Chr9:54317059** | 1 | 0.397 | G | A | *Seita.9G511200*,intorn,  splice site acceptor |
| **Chr9:58943292** | 0.9 | 0.524 | C | T | Intergenic |
