# Supplementary material for: SiSTL2 Is Required for Cell Cycle, Leaf Organ Development, Chloroplast Biogenesis, and Has Effects on C4 Photosynthesis in Setaria italica (L.) P. Beauv
Source: Front Plant Sci. 2018 Jul 30;9:1103. doi: 10.3389/fpls.2018.01103 (PMC6077218; doi:10.3389/fpls.2018.01103)
Supplement: TABLE S9 [file Table_9.DOC]

**Supplementary Table S9. The qRT-PCR primers for validating the RNAseq result**

| \| Gene ID \| Forward primer sequence \| Reverse primer sequence \| \| --- \| --- \| --- \| \| *Seita.J018400* \| ACATCTCTCAGATGCAAGTC \| TACAAGAAGTAGGAGGGGTT \| \| *Seita.6G158500* \| TACTGAGGGGTGATTGGTAT \| GGACAAGCAGGTGTAAAAAG \| \| *Seita.3G184900* \| AAATTTGATAGCACTGTGGC \| TTTTCCTATGTGCCAACTCT \| \| *Seita.3G149000* \| ATATAGATAGTTGGGGTGCG \| GCAAAACCACTCTTGAAACC \| \| *Seita.6G032200* \| GGCAATTTCTCTGTGCCTTA \| CAGGTTGTATGTGTAACAGC \| \| *Seita.4G045900* \| AATTGCCCAAACCCTAACTCC \| GCTTGGATTTGGATCTGGAAG \| \| *Seita.1G352100* \| TCCCTCCTCTATTACTCTCTTC \| GAACTCTTTGAACTTGCGGA \| \| *Seita.6G050100* \| GAGCAGAATGGCAGTATCTT \| CACAAGGACTGAAGGAACAT \| \| *Seita.5G394500* \| TAACTAACGAAACCTGCACC \| AAGCTCTCGAATCATCGAA \| \| *Seita.9G171000* \| GAAGTTGAAGCTCAGTAGGA \| TCTGGACTTGTAGAACTGTG \| \| *Seita.5G005800* \| TTTCAGGGGATTAGAGGAGT \| GCAAATGCGCAATGACC \| \| *Seita.5G352700* \| GCGTTATCTGACTTGTGATG \| ACCAGGTGAGGAATTCTGATA \| \| *Seita.2G283000* \| TTTTCGAGGTTGTTGGTGTG \| TGAGGACTCGAGGGAATTAG \| |
| --- | --- | --- | --- | --- | --- | --- | --- | --- | --- | --- | --- | --- | --- | --- | --- | --- | --- | --- | --- | --- | --- | --- | --- | --- | --- | --- | --- | --- | --- | --- | --- | --- | --- | --- | --- | --- | --- | --- | --- | --- | --- | --- |
|  |
