# Supplementary material for: SiSTL2 Is Required for Cell Cycle, Leaf Organ Development, Chloroplast Biogenesis, and Has Effects on C4 Photosynthesis in Setaria italica (L.) P. Beauv
Source: Front Plant Sci. 2018 Jul 30;9:1103. doi: 10.3389/fpls.2018.01103 (PMC6077218; doi:10.3389/fpls.2018.01103)
Supplement: FIGURE R1 [file Presentation_1.PPT]

## Slide 1
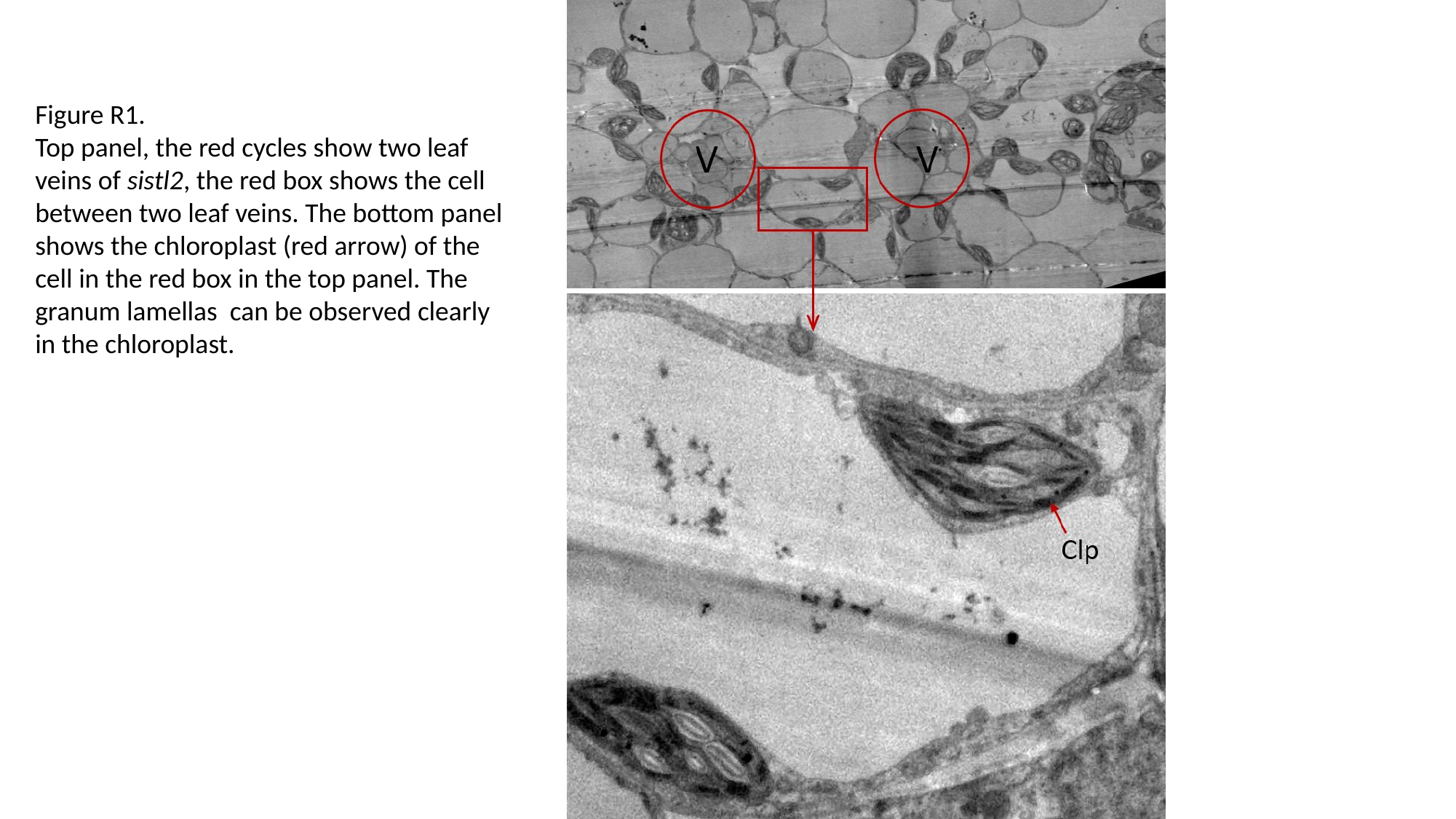

Figure R1.
Top panel, the red cycles show two leaf veins of sistl2, the red box shows the cell between two leaf veins. The bottom panel shows the chloroplast (red arrow) of the cell in the red box in the top panel. The granum lamellas can be observed clearly in the chloroplast.

## Slide 2
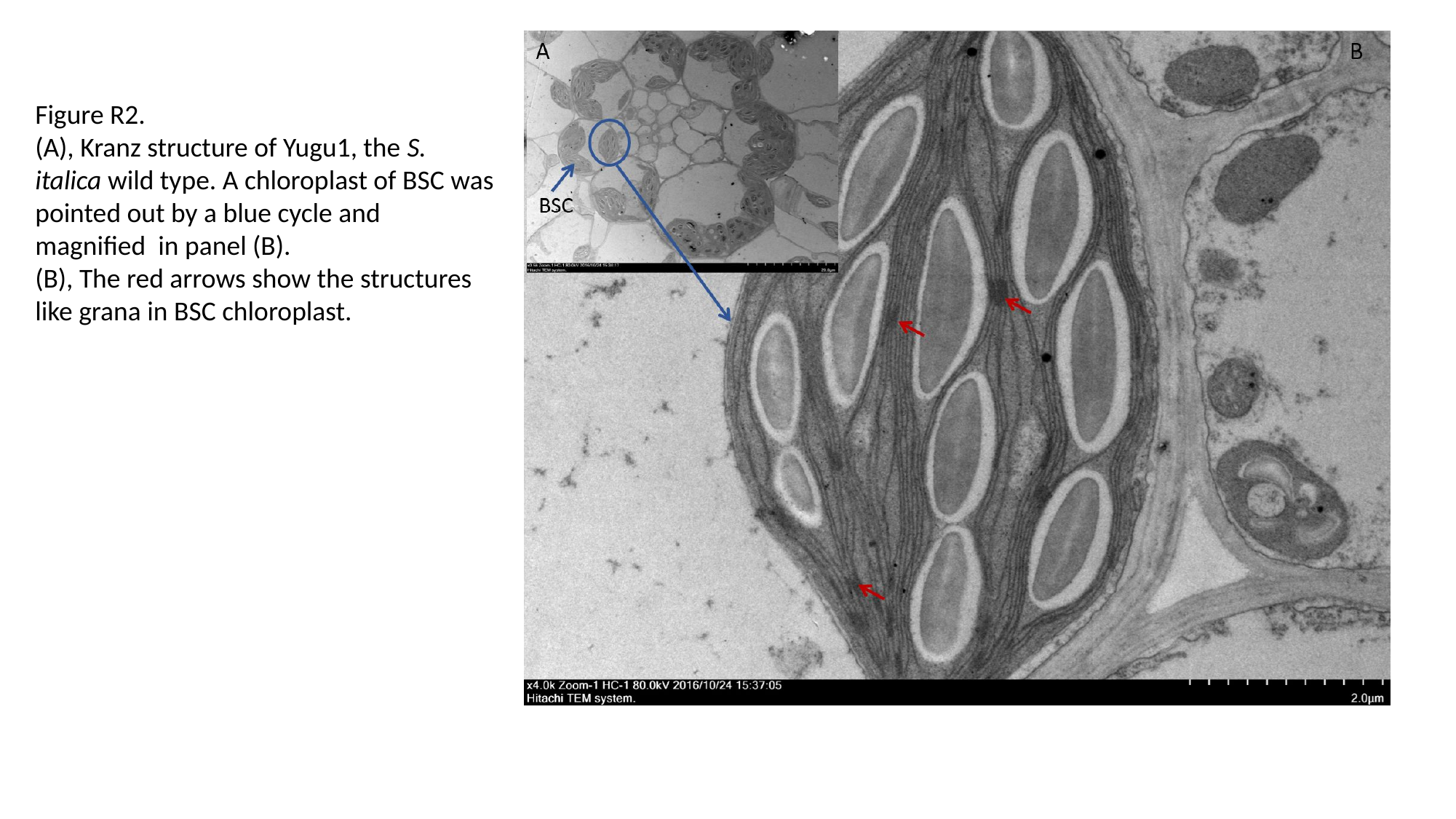

Figure R2.
(A), Kranz structure of Yugu1, the S. italica wild type. A chloroplast of BSC was pointed out by a blue cycle and magnified in panel (B).
(B), The red arrows show the structures like grana in BSC chloroplast.
